# A Treatment for Diseased Pulps

**Published:** 1906-07

**Authors:** 


### A TREATMENT FOR DISEASED PULPS.

Dr. Gillette Hayden recommends the following preparation in the treatment of diseased pulps:

Liquid:

| | |
|---|---|
| Eugenol, | ½ ounce |
| Black's "1, 2, 3," | ¼ ounce |
| Oil of Cloves | 20 drops |
| Oil of cassia | 15 drops |
| Oil of cinnamon | 15 drops |

Powder:
Zinc Oxid.

The liquid and the powder are mixed together to form a stiff paste or cement, which may be used in any case where "toothache" is caused by a diseased but living pulp. By placing a filling of this preparation in the cavity of an aching tooth, an almost immediate cessation of pain is secured, as the medicaments in the formula act as obtunders, at the same time relieving the congested condition. In cases of acute pulpitis, especially where the pulp is exposed by caries, it is not necessary to remove the decay entirely, before placing the filling. The cement is slow to harden and will not stand the force of mastication for a few hours, but it will not wash out, either before or after hardening.

As long as the active agents in the cement exert their influence over the pulp there will be no pain or discomfort to the pattent, and the pulp wil return to als nearly a normal state as is possible under varying conditions.—*Cosmos Periscope.*

